# Value of Early Circulating Tumor Cells Dynamics to Estimate Docetaxel Benefit in Metastatic Castration-Resistant Prostate Cancer (mCRPC) Patients

**DOI:** 10.3390/cancers13102334

**Published:** 2021-05-12

**Authors:** Rebeca Lozano, David Lorente, Isabel M. Aragon, Nuria Romero-Laorden, Paz Nombela, Joaquim Mateo, Alison H. M. Reid, Ylenia Cendón, Diletta Bianchini, Casilda Llacer, Shahneen K. Sandhu, Adam Sharp, Pasquale Rescigno, Teresa Garcés, Maria I. Pacheco, Penelope Flohr, Christophe Massard, Pedro P. López-Casas, Elena Castro, Johann S. de Bono, David Olmos

**Affiliations:** 1 Genitourinary Cancer Traslational Research Group, The Institute of Biomedical Research in Málaga (IBIMA), 29010 Málaga, Spain; rlozano@ext.cnio.es (R.L.); isabel.aragon31@gmail.com (I.M.A.); cllacerp@gmail.com (C.L.); teresa.garces_del_rey@kcl.ac.uk (T.G.); mipacheco@cnio.es (M.I.P.); elena.castro@ibima.eu (E.C.); 2 Spanish National Cancer Research Centre (CNIO), Prostate Cancer Clinical Research Unit, 28029 Madrid, Spain; lorente.davest@gmail.com (D.L.); nuriaromerolaorden@gmail.com (N.R.-L.); mpnombela@cnio.es (P.N.); ycendon@cnio.es (Y.C.); pplopez@cnio.es (P.P.L.-C.); 3 Servicio de Oncología Médica, Hospital Provincial de Castellón, 12004 Castellón de la Plana, Spain; 4 Hospital Universitario La Princesa, 28006 Madrid, Spain; 5 The Institute of Cancer Research, London SW7 3RP, UK; jmateo@vhio.net (J.M.); alison.reid@rmh.nhs.uk (A.H.M.R.); diletta.bianchini@nhs.net (D.B.); Shahneen.Sandhu@petermac.org (S.K.S.); Adam.Sharp@icr.ac.uk (A.S.); Pasquale.Rescigno@icr.ac.uk (P.R.); Penny.Flohr@icr.ac.uk (P.F.); Christophe.MASSARD@gustaveroussy.fr (C.M.); Johann.DeBono@icr.ac.uk (J.S.d.B.); 6 The Royal Marsden NHS Foundation Trust, Sutton, London SM2 5PT, UK; 7 Vall d’Hebron Institute of Oncology (VHIO), 08035 Barcelona, Spain; 8 The Sir Peter MacCallum Department of Oncology, The University of Melbourne, Melbourne, VIC 3000, Australia; 9 Candiolo Cancer Institute, FPO-IRCCS, 10060 Turin, Italy; 10 Department of Medical Oncology, Faculté de Medicine Paris-Sud XI, Institut Gustave Roussy, 94805 Villejuif, France

**Keywords:** circulating tumor cells, biomarkers, metastatic castration-resistant prostate cancer, docetaxel, PSA

## Abstract

**Simple Summary:**

The prognostic role of CTC enumeration in mCRPC patients has been established in several studies, demonstrating a higher prognostic performance than post-treatment changes in PSA levels in patients treated with AR signaling inhibitors, but not taxanes. We carried out a pooled analysis of two prospective studies in mCRPC patients treated with docetaxel. The results of this study showed a greater ability of early changes in circulating tumor cells (CTCs) compared to PSA response endpoints to predict overall survival in metastatic castration-resistant prostate cancer (mCRPC) patients treated with docetaxel. These results encourage the clinical usefulness of CTC enumeration to determine the outcome of mCRPC patients.

**Abstract:**

Circulating tumor cell (CTC) enumeration and changes following treatment have been demonstrated to be superior to PSA response in determining mCRPC outcome in patients receiving AR signaling inhibitors but not taxanes. We carried out a pooled analysis of two prospective studies in mCRPC patients treated with docetaxel. CTCs were measured at baseline and 3–6 weeks post treatment initiation. Cox regression models were constructed to compare 6-month radiographical progression-free survival (rPFS), CTCs and PSA changes predicting outcome. Among the subjects, 80 and 52 patients had evaluable baseline and post-treatment CTC counts, respectively. A significant association of higher baseline CTC count with worse overall survival (OS), PFS and time to PSA progression (TTPP) was observed. While CTC response at 3–6 weeks (CTC conversion (from ≥5 to <5 CTCs), CTC30 (≥30% decline in CTC) or CTC0 (decline to 0 CTC)) and 6-month rPFS were significantly associated with OS (all *p* < 0.005), the association was not significant for PSA30 or PSA50 response. CTC and PSA response were discordant in over 50% of cases, with outcome driven by CTC response in these patients. The c-index values for OS were superior for early CTC changes compared to PSA response endpoints, and similar to 6-month rPFS. Early CTC declines were good predictors of improved outcomes in mCRPC patients treated with docetaxel in this small study, offering a superior and/or earlier estimation of docetaxel benefit in comparison to PSA or rPFS that merits further confirmation in larger studies.

## 1. Introduction

The treatment landscape for metastatic castration-resistant prostate cancer (mCRPC) has rapidly evolved in recent years. Although several drugs have demonstrated an improvement in overall survival (OS) in this setting [1], the optimal treatment sequence has not been established. The development of early treatment response biomarkers is urgently needed to avoid unnecessary exposure to ineffective therapy with undesirable toxicities and to accelerate the process of drug development.

Recommendations for the assessment of treatment response have been developed by the Prostate Cancer Working Group (PCWG) [2]. These are mainly based on computer tomography (CT) or bone scans, prostate-specific antigen (PSA) and clinical deterioration. The evaluation of radiographic response to treatment in advanced prostate cancer is significantly hampered by the fact that over 50% of patients present with bone-only disease, which is non-evaluable by RECIST criteria. Bone scans, on the other hand, do not usually change in the setting of a response and can only define progression after at least 14 weeks have elapsed, due to the potential for spurious flare reactions.

Therefore, PSA remains the cornerstone for monitoring antitumor activity in mCRPC patients with bone-only disease. This biomarker is widely used to make decisions on whether to start or change treatment for prostate cancer, but PCWG3 recommendations establish that PSA progression should not be defined before week 12 because of flare phenomenon or late response [2,3,4]. Although the prognostic significance of PSA as an early biomarker has been consistently reported in patients treated with androgen-receptor signaling inhibitors (ARSIS) [5,6,7,8,9,10], evidence to support the PSA measure as a surrogate for OS is lacking. Due to these and other limitations, many physicians continue to rely on clinical progression to make the decision to switch therapy in these patients [1].

Circulating tumor cells (CTCs) have the potential to improve prognostic and response assessment in advanced prostate cancer. The prognostic value of CTC enumeration in mCRPC patients, both before and during systemic therapy, has been established in several studies [11,12,13,14,15,16]. Baseline “unfavorable” CTC count, defined as ≥5 CTCs/7.5 mL of blood, has consistently been associated with diminished OS. Additionally, post-treatment CTC declines have been associated with improved outcomes in mCRPC patients, with a higher prognostic performance than post-treatment changes in PSA levels [11,14,16].

Currently, PCGW3 recommends the assessment of CTCs as an endpoint for activity in clinical trial, measuring it as frequently as PSA [2]. However, data allowing comparison of the clinical utility of CTCs versus PSA in a chemotherapy-treated population remain scarce. Furthermore, the degree and significance of discordance between CTC and PSA response measures is currently unknown.

We carried out a pooled analysis of two prospective multicenter studies to examine the prognostic value of CTC counts before and after treatment in a cohort of mCRPC patients treated with docetaxel.

## 2. Materials and Methods

### 2.1. Patient Population and Study Procedures

Patients were enrolled either in a single-center prospective study at the Royal Marsden Hospital (RMH) (UK) or in the PROSTAC (Clinical-Trials.gov Identifier: NCT02362620) CTC-substudy conducted at “Hospitales Universitarios Virgen de la Victoria y Regional de Málaga” (Spain). In both studies, patients with mCRPC (serum testosterone ≤50 ng/dL) and an Eastern Cooperative Oncology Group (ECOG) performance status (PS) ≤2 who had a histological diagnosis of prostate adenocarcinoma were eligible if they were going to be treated with standard-of-care docetaxel (75 mg/m^2^ q3w) plus prednisone (5 mg bid) as first- or second-line treatment for mCRPC. In order to enrich for patients with CTC counts >5/7.5 mL, patients in both studies were required to meet at least two of the following criteria associated with high CTC counts (≥5 CTCs) [11]: bone metastases, elevated alkaline phosphatase (ALP > ULN), hemoglobin (Hb) < 10 g/dL, baseline PSA > 150 ng/mL and/or ≥2 prior lines of hormonal therapies. Both studies were approved by the institutional human ethics review boards, and all patients provided written informed consent. The RMH study was conducted between April 2009 and August 2012, and the PROSTAC CTC-substudy between January 2013 and December 2016.

CTCs were measured at baseline within 7 days of starting docetaxel (week 1, day 1). In those patients with CTC counts ≥5/7.5 mL at baseline, CTCs were enumerated again before administering cycle 2 and/or cycle 3 of docetaxel. In addition, optional samples for measuring exploratory biomarkers were obtained during cycle 1 (4 h and/or 24 h post-docetaxel administration). CTC enumeration was performed using the CellSearch™ assay (Menarini Silicon Biosystems Inc., Huntington Valley, PA, USA) [17].

Previous medical history, baseline physical examination, ECOG and laboratory tests, including Hb, ALP, lactate dehydrogenase (LDH), albumin and PSA values were obtained at baseline and with every 3 week cycle. CT scans of the thorax-abdomen-pelvis and Tc-99m-diphosphonate bone-scintigraphs were performed at baseline (within 4 weeks of docetaxel cycle 1, day 1) and repeated every 12 weeks until progression.

### 2.2. Statistical Analyses

There was no formal sample size calculation for this pooled analysis, as in both prospective studies, sample size (RMH *n* = 40 and PROSTAC CTC-substudy *n* = 50) was established for the exploration of different molecular biomarkers in response to docetaxel beyond CTC enumeration. Baseline CTC counts were categorized into three prognostic groups (<5 CTC/7.5 mL, 5–50 CTC/7.5 mL and >50 CTC/7.5 mL of blood) as previously described [11].

Post-treatment CTC response was determined 3 to 6 weeks after docetaxel initiation and was defined as either a ≥30% CTC decline from baseline (CTC30), a conversion from ≥5 CTC/7.5 mL to <5 CTC/7.5 mL (CTC Conversion) or a conversion from ≥1 CTC/7.5 mL to 0 CTC (0CTC). PSA response was determined at week 12 as 30% (PSA30) or 50% (PSA50) decline from baseline. Patients who achieved these thresholds by CTCs and/or PSA were classified as responders.

The Kaplan–Meier method was used to estimate median survival and 95% confidence intervals. Cox proportional hazards models were constructed to explore the association between baseline CTC counts, CTC responses, PSA response endpoints and overall survival (OS), progression-free survival (PFS) or time to PSA progression (TTPP). The performance of the survival models including the different endpoints was assessed with the concordance index (c-index). Analyses were performed using SPSS version 21 (IBM Corp., Armonk, NY, USA) and R version 3.4.1.

## 3. Results

### 3.1. Patient Characteristics

A total of 80 (91.9%) mCRPC patients out of 87 patients screened between both studies (RMH and PROSTAC-CTC) met the eligibility criteria and were included in this analysis (Figure S1).

After a median follow-up of 42.7 months in censored patients, the median OS of the overall population was 19.2 months (95%CI 18.2–25.3), with 70 (87.5%) events. Of those patients, 53 (66.3%) received docetaxel as first-line therapy for mCRPC, and 27 (33.8%) as second-line therapy. Median age was 72.2 years (range 47.6–87.9), 67 patients (83.8%) had bone metastases and 74 (92.6%) had ECOG PS 0–1. Other baseline characteristics are summarized in Table 1.

### 3.2. Baseline CTC Count and Prognosis

Median baseline CTC count was 6.5 cells/7.5 mL (range 0–1266). Twenty patients (25%) had a favorable (<5 CTC/7.5 mL) CTC counts, while 60 patients (75%) had unfavorable counts (>5 CTC/7.5 mL). Of those patients with baseline unfavorable counts, 49 (81.7%) and 11 (18.3%) presented with baseline CTCs counts ≥5–50/7.5 mL or >50/7.5 mL, respectively.

The associations between baseline characteristics and CTC counts are shown in Table 2. Higher CTC counts were associated with poor ECOG PS (*p* = 0.001), higher ALP (*p* = 0.038) and LDH values (*p* < 0.001) and lower hemoglobin levels (*p* < 0.001).

**Figure 1:**
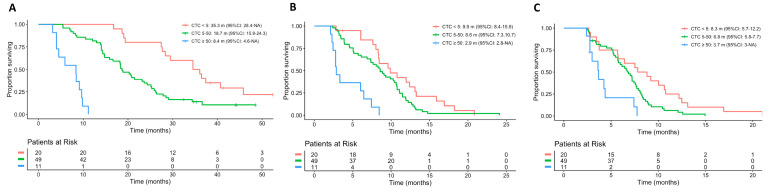
Kaplan–Meier plot for (**A**) overall survival; (**B**) progression-free survival and (**C**) time to PSA progression according to baseline CTC count. Abbreviations: CTC, circulating tumor cell; m, months.

Baseline CTC counts (as a continuous variable) were significantly associated with OS (hazard ratio (HR): 1.02; 95%CI: 1.01–1.03). Patients with CTCs ≥5/7.5 mL experienced a worse OS (HR: 3; 95%CI: 1.7–5.4; *p* < 0.001) than those with CTCs < 5/7.5 mL (Figure 1, Table 3). Similarly, patients with CTCs >50/7.5 mL had worse outcomes than patients with 5–50 or <5 CTCs/7.5 mL (Figure 1, Table 3).

### 3.3. CTC and PSA Decline as Response Measures

Of the 80 eligible patients, 52 (65%) had ≥5/7.5 mL CTCs at baseline and had a second blood collection, and were eligible for response analysis. Compared to baseline, 34 patients (65.4%) experienced a ≥30% decline in CTC count (CTC30), 27 patients (51.9%) had a conversion from ≥5 CTCs at baseline to <5 CTCs (CTC Conversion) and 8 (15.4%) patients experienced a decline to 0 CTCs (CTC0). Of these 52 patients, 32 (61.5%) and 22 (42.3%) experienced a 30% and 50% PSA decline, respectively.

**Figure 2:**
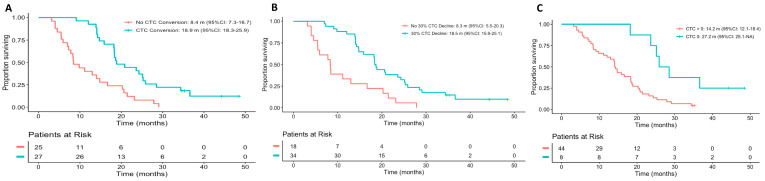
Kaplan–Meier plots for overall survival according to CTC measured as (**A**) CTC conversion from ≥5 CTC/7.5 mL to <5 CTC/7.5 mL; (**B**) 30% CTC decline from baseline and (**C**) conversion from ≥1 CTC/7.5 mL to 0 CTC (CTC0). Abbreviations: CTC, circulating tumor cell; m, months.

CTC decline ≥30% (CTC30) from baseline (Yes 18.5 vs. No 8.3 months, *p* < 0.001), CTC conversion (Yes 18.9 vs. No 8.4 months, *p* < 0.001) and CTC0 decline (Yes 27.2 vs. No 14.2 months, *p* = 0.005) responses were all associated with an increased OS (Figure 2). However, neither PSA by either 30% or 50% declines were significantly associated with survival (Table 4).

CTC response endpoints had a consistently higher discriminating ability than PSA response endpoints in Cox-PH models. CTC conversion (c-index: 0.664), CTC30 (c-index: 0.644) and CTC0 (c-index: 0.602) had higher c-indices than PSA50 (c-index: 0.594) or PSA30 (c-index: 0.563) (Table 4). Differences in time-dependent ROC AUCs are represented in Figure S2.

### 3.4. Concordance between CTC, PSA Response and rPFS

We then evaluated the degree of concordance between a 30% CTC decline and a 30% PSA response. In most cases (22 cases, 42.3%), CTC and PSA responses were concordant. In 12 (23.1%) cases, however, patients experienced a CTC response without a PSA response. On the other hand, 8 (15.4%) patients experienced a PSA response without a CTC response, and 10 (19.2%) patients experienced neither a PSA nor a CTC response.

Median OS was similar in patients with a concordant response in both PSA and CTCs (19.5 months; 95%CI: 15.9–28.5 months), and in patients with a CTC response without PSA response (18.5 months; 95%CI: 13.9–NA). Patients with a PSA response but no CTC response had a numerically lower survival (9.8 months) than those with a CTC response but no PSA response, although this difference was not statistically significant (*p* = 0.667). Patients with no response in terms of either PSA or CTCs (concordant non-response) had the lowest survival (7.1 months) (Table S1).

We then compared the performance of 6-month radiographic progression-free survival (rPFS) to the different CTC response measures. Of 52 patients that reached the 6-month landmark survival point, 49 were included. Among the 33 patients who experienced an early CTC decline greater than 30%, 26 (78.8%) were radiographic progression-free at 6 months, while only 7 (21.2%) experienced an rPFS event. Six-month rPFS was significantly associated with OS (20.3 vs. 7.5 months; HR 2.9; 95%CI: 1.52–5.48; *p* < 0.001), but the OS discriminating ability of 6-month rPFS (c-index: 0.649) was not superior to that of 30% CTC decline (c-index: 0.644) or CTC conversion (c-index: 0.664).

## 4. Discussion

The prognostic value of baseline CTC enumeration in mCRPC patients has been well documented in post-hoc analyses from several clinical trials including patients treated both with chemotherapy [11,12,18,19,20] and ARSIS [13,21]. However, data from patients on chemotherapy come from subjects treated in combination trials of docetaxel with lenalidomide, atrasentan or other drugs [11,19,20].

Here, we report the combined results of two prospective studies carried out in cohorts of patients treated only with docetaxel as first- or second-line therapy in routine clinical practice. In line with the current evidence, we observed an association of high baseline CTC counts with poor prognostic factors and features of high tumor burden (elevated ALP, LDH, poor ECOG PS or low hemoglobin), although both studies were precisely designed to enrich patients with these characteristics and high CTC counts.

Furthermore, in line with results from previous studies, we observed an association between higher baseline CTC and poor outcome, not only in terms of OS but also PFS (Figure 1). Our results support the premise that it is preferable to consider changes in CTC counts as a continuous variable, rather than a simple dichotomy between “favorable” and “unfavorable” CTCs, and that patients with very high CTC counts have a particularly adverse prognosis. In our study, patients with >50 CTCs at baseline had almost four times shorter median OS compared with those patients with <5 CTCs, and half the median OS observed in patients with 5–50 CTCs (Figure 1A), supporting previously published evidence [11].

Previous studies have also shown that changes in CTCs during treatment can be a reliable indicator of response, demonstrating that early decline in CTC counts after treatment is associated with improved outcomes in mCRPC patients [11,12,14]. However, there have not been consistent criteria for the definition of a response to treatment.

Although PCWG3 criteria recommend evaluating the rate of conversion from unfavorable to favorable CTC counts as a measure of response in clinical trials [2], by this strategy, as many as 60% of treatment-naïve, minimally symptomatic patients could be ineligible for response assessments [16]. Alternative response criteria, such as 30% declines from baseline or conversion from detectable (≥1CTC) to undetectable CTC counts (CTC0), were proposed in other studies. In a pooled analysis of five clinical trials in mCRPC patients treated with ARSIS and targeted therapy (but no chemotherapy), the CTC0 endpoint had a better performance than all other endpoints in the prognostic models [16]. In our study, however, c-index values were higher for the CTC conversion (0.665) and CTC30 (0.644) than the CTC0 (0.602) endpoints. This may be due to the greater baseline CTC counts in our study; all of our patients that had follow-up CTC counts had ≥5 CTCs at baseline. On the other hand, a greater performance of these endpoints in chemotherapy-treated patients, as opposed to ARSIS-treated patients, cannot be ruled out.

PSA is the most widely used response biomarker in the day-to-day care of patients with advanced prostate cancer. Studies have found a significant association between 30% or 50% declines and OS in several clinical trials [9,10]. In our study, however, neither 30% nor 50% PSA declines were associated with survival. One must take into account that PSA is a pharmacodynamic biomarker that is ultimately related with the activity of the androgen receptor (AR) pathway. PSA level changes might be more useful for monitoring responses in patients treated with drugs that directly target AR pathways, such as abiraterone acetate or enzalutamide.

In our study, CTC-based endpoints had consistently higher c-index values (from 0.602 to 0.664) than PSA-based response endpoints (from 0.563 to 0.594). This is in line with results from previous studies that have shown that CTC count is a better predictor of survival than post-treatment changes in PSA [11,12,16]. Our results suggest that in cases with discordant PSA and CTC responses, response (or lack thereof) by CTCs is a more reliable indicator of outcome than PSA.

A number of limitations must be acknowledged. First, our small sample size, with 52 patients eligible for CTC response analysis, may limit the interpretation of the results, especially in comparison with other studies, such as that reported by Heller and colleagues [16], performed in over 5000 advanced prostate cancer patients. Secondly, only patients with baseline CTC counts ≥5/7.5 mL were selected for response evaluation. This may be a potential source of bias, especially when comparing the performance of the different CTC response endpoints.

## 5. Conclusions

In conclusion, baseline CTC enumeration is a good predictor of survival in patients treated with docetaxel as first- or second-line therapy. Likewise, in our cohort of patients, early CTC decline after treatment was demonstrated to be a better predictor of survival, with better performance than PSA response endpoints. Despite the limitations of our small study, these results suggest CTC response might serve as an early biomarker of treatment response, avoiding unnecessary exposure to failure therapies and undesirable toxicities. Further prospective clinical trials incorporating CTC as an endpoint for activity are needed.

## Figures and Tables

**Table 1:** Patient characteristics at baseline and treatments.

Characteristics	*n* (%)
Docetaxel line	
First-line	53 (66.2%)
Second-line	27 (33.8%)
Age at diagnosis (years) Median (range)	72.2 (47.6–87.9)
Histology grade	
Gleason < 8	53 (66.2%)
Gleason ≥ 8	27 (33.8%)
M1 at diagnosis	
No	35 (43.8%)
Yes	45 (56.2%)
PSA (ng/dL) Median (range)	60.6 (2.2–1428)
Metastasis site	
Bone	67 (83.8%)
Lymph node	41 (51.2%)
Visceral	12 (15%)
Performance status	
ECOG 0	29 (36.2%)
ECOG 1	45 (56.2%)
ECOG 2	6 (7.5%)
ALP > ULN	42 (52.5%)
LDH > ULN	32 (40%)
Hemoglobin < 10 g/dL	7 (8.8%)
Albumin < 3.5 g/dL	13 (16.2%)
Prior treatment	
None	53 (66.2%)
Abiraterone	20 (25%)
Enzalutamide	7 (8.8%)
Median time from diagnosis to mCRPC—months (range)	31.6 (1.8–229)
Median time from continuous ADT to mCRPC—months (range)	27.9 (4.9–205.6)

Abbreviations: PSA, prostate-specific antigen; ECOG, Eastern Cooperative Oncology Group; ALP, alkaline phosphatase; LDH, lactate dehydrogenase; mCRPC, metastatic castration-resistant prostate cancer; ADT, androgen deprivation therapy.

**Table 2:** Associations between CTC count and baseline characteristics of patients.

Characteristics	*n*	CTC/7.5 mL		*p*-Value
		Median	Range	
CTC count at baseline	80	6.5	0–1266	-
Docetaxel line				0.3
First-line	53	6	0–891	
Second-line	27	9	0–1266	
Age (years)				0.13
<Median	40	6	0–567	
≥Median	40	7	0–1266	
Histology				0.81
Gleason < 8	27	6	0–891	
Gleason ≥ 8	53	7	0–1266	
M1 at diagnosis				0.27
Yes	45	9	0–1266	
No	35	6	0–567	
PSA (ng/dL)				0.76
<Median	40	5	0–891	
≥Median	40	8.5	0–1266	
Metastasis site				
Bone	67	7	0–1266	0.35
Lymph node	41	7	0–891	0.4
Visceral	12	10	0–891	0.87
ECOG				0.001
ECOG 0	29	5	0–51	
ECOG 1	45	9	0–567	
ECOG 2	6	14.5	0–1266	
ALP				0.038
Normal	38	5	0–56	
Elevated (>ULN)	42	9	2–1266	
LDH				0.007
Normal	48	5	0–98	
Elevated (>ULN)	32	10	5–1266	
Hemoglobin				<0.001
≥10 g/dL	73	6	0–567	
<10 g/dL	7	7	0–1266	
Albumin				0.07
≥3.5 g/dL	66	6	0–1266	
<3.5 g/dL	13	19	6–891	

Abbreviations: CTC, circulating tumor cell; PSA, prostate-specific antigen; ECOG, Eastern Cooperative Oncology Group; ALP, alkaline phosphatase; LDH, lactate dehydrogenase; ULN, upper limit of normal; mCRPC, metastatic castration-resistant prostate cancer; ADT, androgen deprivation therapy.

**Table 3:** Association of survival outcomes and baseline CTC count.

Overall Survival			
Category	Median	HR (95%CI)	*p*-Value
<5 CTCs	35.3 (28.4–NA)	1	1
5–50 CTCs	18.7 (15.9–24.3)	2.69 (1.47–4.93)	0.0014
>50 CTCs	8.4 (4.5–NA)	40.4 (13.6–119.6)	<0.0001
Progression-Free Survival (composite endpoint)			
<5 CTCs	9.9 (8.4–15.9)	1	1
5–50 CTCs	8.6 (7.4–10.7)	1.66 (0.96–2.88)	0.0691
>50 CTCs	2.9 (2.8–NA)	8.13 (3.56–18.57)	<0.0001
Time to PSA Progression			
<5 CTCs	8.3 (5.7–12.2)	1	1
5–50 CTCs	6.8 (5.8–7.7)	1.96 (1.12–3.45)	0.0187
>50 CTCs	3.7 (3–NA)	5.35 (2.31–12.37)	0.0001

Abbreviations: CTC, circulating tumor cell; PSA, prostate-specific antigen; NA, not available.

**Table 4:** Median overall survival, hazard ratios and c-index values for CTC and PSA response endpoints.

CTC/PSA Response	*n* (%)	Median OS (Months)		HR (95%CI)	*p*-Value	c-Index
		Yes	No			
CTC30	34 (65.4)	18.5	8.3	0.33 (0.18–0.61)	0.0004	0.6436
CTC Conv	27 (51.9)	18.9	8.4	0.33 (0.18–0.60)	0.0003	0.6645
CTC0	8 (15.4)	27.2	14.2	0.26 (0.1–0.67)	0.0053	0.6018
PSA30	32 (62.7)	18.3	13.4	0.72 (0.40–1.29)	0.2692	0.5631
PSA50	22 (43.1)	19.3	13.4	0.68 (0.38–1.20)	0.1808	0.5946

Abbreviations: OS, overall survival; CTC, circulating tumor cells; PSA, prostate specific antigen. Yes and No: Patients classified as responders or non-responders, respectively, according to the different definitions for CTC or PSA response.

## Data Availability

Limited data presented in this study are available on request from the corresponding author. The full study dataset are not publicly available at the present due to ongoing planned analysis.

## Supplementary Materials

The following are available online at https://www.mdpi.com/article/10.3390/cancers13102334/s1, Figure S1: Study flowchart. Figure S2: Time-dependent ROC areas under the curve (AUCs) of the CTC response endpoints, PSA response endpoints and 6-month radiographic progression-free survival. Table S1: Concordance between CTC and PSA response endpoints.
