# Supplementary material for: Value of Early Circulating Tumor Cells Dynamics to Estimate Docetaxel Benefit in Metastatic Castration-Resistant Prostate Cancer (mCRPC) Patients
Source: Cancers (Basel). 2021 May 12;13(10):2334. doi: 10.3390/cancers13102334 (PMC8151844; doi:10.3390/cancers13102334)
Supplement: Supplementary file 1 [file cancers-13-02334-s001.zip › cancers-1162000-supplementary.pdf]

cancers

*Supplementary*

# Value of Early Circulating Tumor Cells Dynamics to Estimate Docetaxel Benefit in Metastatic Castration-Resistant Prostate Cancer (mCRPC) Patients

**Rebeca Lozano et al.**

## Supplementary Statistical Methods

OS was defined as the time from blood collection to the date of death or the last follow-up. Progression-free survival (PFS) was defined as the time from blood collection to disease progression based on a composite end-point including: i) radiographic progression according to PCWG2 and/or RECIST 1.1 criteria, ii) clinical progression defined by investigator criteria or iii) death. Time to PSA progression (TTPP) was defined as the time from blood collection to PSA progression, according to PCWG2 criteria.

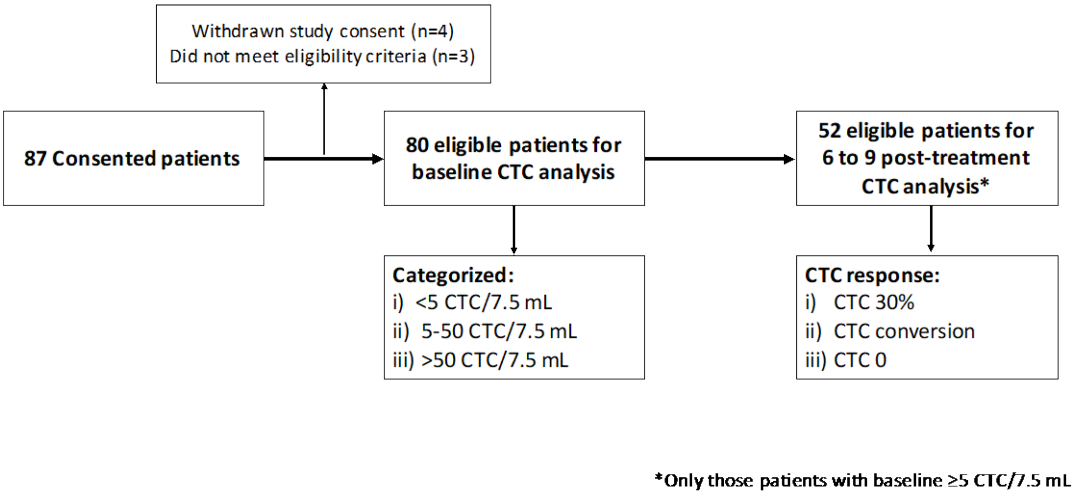


**Figure S1.** Study flowchart.

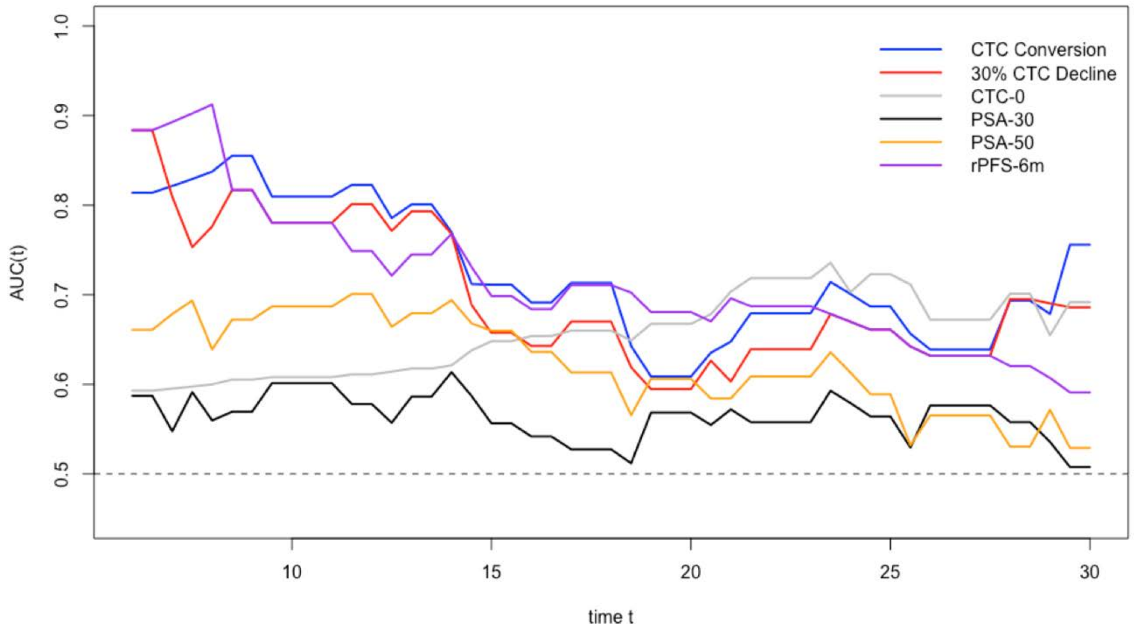


**Figure S2.** Time-dependent ROC areas under the curve (AUCs) of the CTC response endpoints, PSA response endpoints and 6-month radiographic progression-free survival.

**Table S1.** Concordance between CTC and PSA response endpoints.

| PSA 30 Response | CTC 30% Response | N (%) | Median OS (months) | HR (95% CI) | p-value |
|---|---|---|---|---|---|
| No | No | 8 (15.4) | 7.1 (4.5-NA) | 5.12 (2.14-12.24) | <0.001 |
| No | Yes | 12 (23.1) | 18.5 (13.9-NA) | 1.18 (0.55-2.55) | 0.667 |
| Yes | No | 10 (19.2) | 9.8 (5.49-NA) | 2.53 (1.15-5.53) | 0.020 |
| Yes | Yes | 22 (42.3) | 19.5 (15.9-28.5) | - | - |
| **PSA 30 Response** | **CTC Conversion** | **N (%)** | **Median OS (months)** | **HR (95% CI)** | **p-value** |
| No | No | 10 (19.2) | 7.8 (4.5-NA) | 6.40 (2.67-15.34) | <0.001 |
| No | Yes | 10 (19.2) | 18.8 (14.1-NA) | 1.12 (0.47-2.67) | 0.801 |
| Yes | No | 15 (28.8) | 14.0 (7.8-21.5) | 2.45 (1.17-5.14) | 0.018 |
| Yes | Yes | 17 (32.7) | 23.6 (18.2-34.4) | - | - |
| **PSA 30 Response** | **CTC 0 Response** | **N (%)** | **Median OS (months)** | **HR (95% CI)** | **p-value** |
| No | No | 19 (36.5) | 13.0 (8.4-20.8) | 4.37 (1.45-13.15) | 0.009 |
| No | Yes | 1 (1.9) | 25.9 (NA-NA) | 1.82 (0.20-16.48) | 0.592 |
| Yes | No | 25 (48.1) | 14.8 (14-20.6) | 4.08 (1.40-11.91) | 0.009 |
| Yes | Yes | 7 (13.5) | 28.5 (23.6-NA) | - | - |
| **PSA 50 Response** | **CTC 30% Response** | **N (%)** | **Median OS (months)** | **HR (95% CI)** | **p-value** |
| No | No | 13 (25) | 8.3 (4.5-NA) | 4.53 (2.07-9.9) | <0.001 |
| No | Yes | 17 (32.7) | 18.4 (14.1-28.5) | 1.07 (0.52-2.2) | 0.848 |
| Yes | No | 5 (9.6) | 16.7 (7.8-NA) | 1.84 (0.66-5.08) | 0.241 |
| Yes | Yes | 17 (32.7) | 20.6 (18.2-29.1) | - | - |
| **PSA 50 Response** | **CTC Conversion** | **N (%)** | **Median OS (months)** | **HR (95% CI)** | **p-value** |
| No | No | 16 (30.8) | 7.8 (5.5-13) | 5.76 (2.54-13.08) | <0.001 |
| No | Yes | 14 (26.9) | 18.8 (14.7-NA) | 0.94 (0.41-2.14) | 0.879 |
| Yes | No | 9 (17.3) | 16.7 (14-NA) | 1.70 (0.71-4.10) | 0.236 |
| Yes | Yes | 13 (25) | 23.6 (18.2-NA) | - | - |
| **PSA 50 Response** | **CTC 0 Response** | **N (%)** | **Median OS (months)** | **HR (95% CI)** | **p-value** |
| No | No | 27 (51.9) | 11.1 (8.4-18.4) | 4.20 (1.34-13.14) | 0.013 |
| No | Yes | 3 (5.8) | 28.5 (25.9-NA) | 0.71 (1.13-3.93) | 0.699 |
| Yes | No | 17 (32.7) | 18.3 (14.8-25.3) | 2.65 (0.83-8.50) | 0.102 |
| Yes | Yes | 5 (9.6) | 25.1 (23.6-NA) | - | - |

Abbreviations: PSA, prostate-specific antigen; CTC, circulating tumor cell; OS, overall survival; HR, hazard ratio; PSA30, PSA response determined as 30% decline from baseline; PSA50, PSA response determined as 50% decline from baseline. NA, not available.
